# Endothelial glycocalyx shedding and vascular permeability in severely injured trauma patients

**DOI:** 10.1186/s12967-015-0481-5

**Published:** 2015-04-12

**Authors:** Elaheh Rahbar, Jessica C Cardenas, Gyulnar Baimukanova, Benjamin Usadi, Roberta Bruhn, Shibani Pati, Sisse R Ostrowski, Pär I Johansson, John B Holcomb, Charles E Wade

**Affiliations:** Department of Surgery, Center for Translational Injury Research, University of Texas Health Science Center, Houston, TX USA; Department of Biomedical Engineering, Wake Forest University, 575 N. Patterson Ave, Suite 120, Winston-Salem, NC 27101 USA; Blood Systems Research Institute, San Francisco, CA USA; Department of Laboratory Medicine, University of California San Francisco, San Francisco, CA USA; Capital Region Blood Bank, Copenhagen University Hospital, Rigshospitalet, Copenhagen, Denmark

**Keywords:** Glycocalyx, COP, Hyaluronic acid, Syndecan-1, Permeability, Trauma, Thrombin

## Abstract

**Background:**

The endothelial glycocalyx layer (EGL) is a key regulator of vascular permeability, cell adhesion, and inflammation. The EGL is primarily composed of syndecan-1, hyaluronic acid (HA), heparan sulfate (HS) and chondroitin sulfate (CS). While many studies have observed increased shedding of syndecan-1 during hemorrhagic shock, little is known about the shedding of other EGL components, and their effects on altered permeability and coagulation. We characterized shedding of all four primary components of the EGL, as well as the plasma’s effect on permeability and thrombin generation in a cohort of trauma patients.

**Methods:**

Plasma samples were collected from 5 healthy consented volunteers and 22 severely injured trauma patients upon admission to the emergency department. ELISA assays were performed to quantify shed HA, HS, CS and syndecan-1 in plasma. A colloid osmometer and Electric Cell-substrate Impedance Sensing (ECIS) system were used to measure plasma colloid osmotic pressure (COP) and cell permeability, respectively. Thrombin generation was measured using a calibrated automated thrombogram (CAT). Initial vital signs, routine laboratory values, and injury severity scores (ISS) were recorded. Non-parametric statistical tests were used to compare differences between groups.

**Results:**

We observed increased shedding of all four proteins in trauma patient plasma compared to healthy controls: 31.7 vs. 21.2 U/L of CS, 175.8 vs. 121.9 ng/ml of HS, 946.7 vs. 618.6 ng/ml of HA and 245.8 vs. 31.6 ng/ml of syndecan-1 (all p < 0.05). Patients with low plasma COP (≤16 mmHg) had significantly increased syndecan-1 and HA compared to those with normal COP, which corresponded to increased cell permeability via ECIS. CS and HS did not vary between COP groups. Lastly, patients with low COP displayed reduced peak thrombin generation of less than 250 nM on average (p < 0.05).

**Conclusions:**

Glycocalyx components were shed more in trauma patients compared to healthy controls in this cohort. However, only syndecan-1 and HA shedding were significantly higher in patients with reduced plasma COP. Thrombin generation was impaired in patients with low plasma COP. These data suggest that low plasma COP correlates well to glycocalyx degradation and thrombin loss following trauma, which consequently affect permeability and coagulation.

## Background

Hemorrhagic shock is the leading cause of mortality in both civilian and military populations. Nearly 50% of hemorrhagic deaths occur within 3–6 hours of injury [1]. Many of these deaths are considered potentially preventable with appropriate care and resuscitation. Recent efforts in resuscitation for massively injured patients have focused on not only restoring the blood volume lost, but also ameliorating the inflammatory and coagulopathic responses, vascular permeability and endothelial dysfunction [2-4]. As a result, a number of biomarkers for coagulation and endothelial integrity have emerged. Endothelial dysfunction and vascular permeability (i.e. “capillary leak”) have been associated with increased morbidity and mortality; in particular, sepsis, multi-organ failure and hemorrhagic shock [5,6].

The vascular endothelium is protected by a thin layer of cell-bound proteoglycans and glycoproteins, commonly referred to as the endothelial glycocalyx layer (EGL) [7-12]. The major components of the EGL are syndecans, hyaluronic acid (HA), chondroitin sulfate (CS) and heparan sulfate (HS) [7-12]. The EGL plays a critical role in maintaining vascular integrity by serving as a (1) barrier to the transport of proteins and fluids, therefore preventing “capillary leak”, (2) mechano-transducer of pressure and shear stress, and (3) regulator of inflammatory cell adhesion and infiltration [7-12]. Furthermore, the EGL has been shown to serve as a significant binding site for antithrombin III (ATIII), tissue factor pathway inhibitor, vascular endothelial growth factor (VEGF), fibroblast growth factor (FGF), and lipoprotein lipase [10-12].

Once the EGL is disrupted, endothelial cells are exposed consequently allowing for the adhesion of platelets, leukocytes and other neutrophil-endothelial cell interactions [8,12,13]. Several studies have shown increased shedding of syndecan-1, catecholamines and cytokines following hemorrhagic shock in both *in vitro* and rodent models [3,14-21]. Shedding of syndecan-1 has been shown to be ameliorated with freshly frozen plasma (FFP) resuscitation, suggesting that FFP may play a role in the partial restoration of the EGL [3,15,18]. More recently, Torres et al. have shown restoration of syndecan-1 and heparan sulfate levels with FFP and fresh whole blood resuscitation in an animal model of hemorrhagic shock, which also correlated to EGL repair [19-21]. Resuscitation with FFP has also been associated with modulation of syndecan-1 shedding in human subjects [5]. Trauma patients who had high levels of shed syndecan-1 also demonstrated higher levels of IL-10, an inflammatory cytokine [5]. Chappell et al. observed significant shedding of the EGL with ischemic injury and preservation of the EGL with ATIII treatment [14]. Thus, the EGL may play a critical role in the inflammatory and coagulopathic responses post-injury.

In the case of hemorrhagic shock and injury, there is also a substantial hemodynamic response. As the patient becomes hypotensive, there is a reduction in hydrostatic and plasma colloid osmotic pressures (COP, also referred to as oncotic pressure), which affects the flux of fluid and solutes between the vascular and interstitial compartments as dictated by the revised Starling principle [22,23]. Plasma COP is the pressure exerted by large molecules on the semi-permeable membranes between the capillary and interstitial spaces. We have previously shown that reduced plasma COP is associated with injury severity and increased shedding of syndecan-1, which consequently translated into increased need of transfusions and resuscitation [24]. In this study we conducted a secondary analysis of prospectively collected data where we hypothesized that low plasma COP would further correlate to the shedding of the four major EGL components: HA, HS, CS and syndecan-1, and subsequently result in increased cell permeability and hemostatic dysfunction which are associated with worse patient outcomes.

## Methods

### Sample collection

This prospective observational study was conducted under an approved IRB (Universal Study, HSC-GEN-12-0059), which included all adult trauma patients (≥16 years) at the highest level of activation at Memorial Hermann Hospital Texas Medical Center (MHH-TMC). Pregnant women and prisoners were excluded from the study. IRB approval was obtained for delayed consent given the nature of trauma patients, therefore consent was obtained from the patient or their legally authorized representative within 48–72 hours of admission, or as soon as possible. If patients were discharged within 24 hours of admission, a waiver of consent was obtained. Blood was collected from each patient in citrated tubes on admission. Samples from patients who refused consent were destroyed. Blood was also collected from healthy volunteers to serve as a control group.

### Osmolality, oncotic pressure and serum protein

Blood samples were centrifuged and plasma was stored for future use at −80°C. Osmolality and COP (i.e. oncotic pressure) were measured from fresh plasma at room temperature as previously described by Rahbar et al. [24]. Briefly, plasma samples were run in duplicate with the Wescor Vapro Osmometer (model 5520, Logan, UT) and Wescor Colloid Osmometer (model 4420, Logan, UT), respectively to obtain osmolality and oncotic pressure readings. Based on our previous study, plasma COP < 16 mmHg was used as the cut-off for the “low COP” group [24]. This cut-off was determined by calculating two standard deviations away from the average normal COP levels from healthy volunteers. Serum protein was measured from fresh plasma at room temperature using a clinical refractometer (RHC-200/ATC, C&A Scientific, Manassas, VA).

### Thrombin generation assay

Thrombin generation was measured using calibrated automated thrombogram (CAT; Thrombinoscope, Maastricht, Netherlands) in platelet-poor plasma (PPP), as previously described by Cardenas et al. [25]. All samples were run in duplicate in a 96-well plate and calibrated using 20 μL of α2-macroglobulin/thrombin complex. Each thrombogram curve results in the following parameters: lag time (time to initiation of thrombin formation; min), endogenous thrombin potential (ETP; the area under the curve; nM*min), peak (maximum thrombin concentration; nM), and “time to peak” (ttPeak; min).

### Enzyme-linked immunosorbant assays

Commercial enzyme-linked immunosorbant assays (ELISAs) for soluble syndecan-1, chondroitin sulfate, heparan sulfate and hyaluronic acid were performed to quantify levels of endothelial glycocalyx component degradations (Syndecan-1: Abcam, Cat. No. ab46506, Cambridge, MA; Heparan Sulfate: Biotang, Cat. No. HU8718, Lexington, MA; Chondroitin Sulfate: Biotang, Cat. No. HU8720, Lexington, MA; Hyaluronic Acid: R&D Systems, Cat. No. DHYAL0, Minneapolis, MN). Adrenaline and noradrenaline were measured in uniplicate in plasma by commercially available ELISA kits (2-CAT ELISA; Labor Diagnostica Nord GmbH & Co KG, Nordhorn, Germany; lower limit of detection [LLD]) 10 pg/mL (adrenaline; normal reference, < 100 pg/mL) and 50 pg/mL (noradrenaline; normal reference, < 600 pg/mL), respectively [16]. Frozen citrated plasma samples were used for all ELISA assays, except for measuring HA and catecholamines, which used frozen plasma stored in Ethylenediaminetetraacetic acid (EDTA). All other samples were run in duplicate. Samples that were over the detection range of the assay were diluted and rerun as needed.

### Endothelial cell permeability

Cell barrier function was assessed by measuring trans-endothelial electrical resistance (TEER) in real time using the Electric Cell-substrate Impedance Sensing (ECIS® 1600, Applied BioPhysics, Troy, NY) system as described by Giaever and Keese [26,27]. Briefly, human umbilical vein endothelial cells (HUVECs) were grown to confluence on L-cysteine reduced, Fibronectin-coated 96W10E electrodes (Applied BioPhysics, Troy, NY) in endothelial growth medium (EGM-2; Lonza Inc., Walkersville, MD), and serum starved for one hour in endothelial basal medium (EBM-2; Lonza Inc., Walkersville, MD) before treatment. Cells were treated with 10% of the frozen plasma in 200 μL of EBM-2 medium. Monolayer resistance was recorded at 4 kHz over eight-minute intervals for 24 hours. A change in TEER across the cell monolayer indicated increased or decreased paracellular permeability.

#### Analysis of ECIS data

For analysis of changes in barrier function, TEER measurements at 4 kHz were averaged over the first five available post-treatment time points. Mean TEER values were then computed for each plasma sample (trauma patients and healthy volunteers). Normalized TEER values were also calculated by dividing each well by its baseline value (prior to plasma sample addition).

### Clinical parameters

Clinical laboratory results from the time of admission were collected into a database for the consented patients. This included: admission vital signs, complete blood count test results, pH, base excess, clinical coagulation tests including prothrombin time (PT), activated partial thromboplastin time (aPTT), international normalized ratio (INR), pre-hospital fluids, 24 hour blood transfusions (including packed red blood cells (RBCs), freshly frozen plasma (or thawed plasma) and apheresis platelets), 24 hour crystalloid infusions, complications, injury severity score (ISS) and patient outcomes. Total blood units at 24 hours were calculated as the cumulative number of units of blood products transfused within the first 24 hours, using the same conversions as previously published by Rahbar et al. [28]. Patient demographics such as age, gender, race, and mechanism of injury were documented.

### Statistical analysis

All statistical analyses were conducted with commercially available software, STATA (12.1, College Station, TX). Non-parametric tests, including Kruskal-Wallis and Chi-square tests, were used to assess differences between Low and Normal COP groups. Median and inter-quartile (IQR) values are reported throughout. Multiple comparisons were made to determine differences between healthy controls and the Low vs. Normal COP groups. No corrections for multiple comparisons were performed. Statistical significance was set at the 5% level. A multivariable linear regression for permeability was developed while adjusting for age, gender, base excess, injury severity and pre-hospital fluids. A negative binomial regression was developed to assess the incidence risk ratio (IRR) of low plasma COP and the shedding of glycocalyx components on blood transfusion requirements, while adjusting for the aforementioned variables used in the multivariable linear regression.

## Results

A total of 22 trauma patient plasma samples and 5 healthy volunteer plasma samples were used to characterize the glycocalyx component shedding and their effect on endothelial cell permeability. The overall patient demographics were 73% male, median age of 44 years, median ISS of 22, base excess of −2 (−3,0) and an overall in-hospital mortality rate of 36% (Table 1). Patients in the low COP group were more severely injured as demonstrated by the higher ISS, had worse pH and base excess levels and received more blood transfusions within 24 hours compared to the normal COP group (Table 1). These patient demographics and characteristics are consistent with our previously published work [24].

**Table 1:** **Patient demographics, admission vitals, resuscitation volumes and outcomes**

	**Overall (N = 22)**	**Normal COP (N = 11)**	**Low COP (N = 11)**	**p-value**
**Age (yrs)**	44 (31, 54)	46 (31, 56)	41 (29, 51)	0.74
**Male, n (%)**	16 (73%)	8 (73%)	8 (73%)	0.69
**Penetrating, n (%)**	5 (23%)	1 (9%)	4 (36%)	0.22
**SBP (mmHg)**	122 (108, 140)	136 (119, 151)	119 (93, 123)	0.09
**DBP (mmHg)**	67 (54, 80)	75 (59, 80)	60 (51, 74)	0.17
**HR (bpm)**	99 (79, 116)	98 (90, 116)	99 (78, 126)	0.95
**RR (bpm)**	17 (15, 20)	16 (0, 18)	18 (15, 22)	0.28
**GCS**	9 (3, 15)	8 (3, 15)	10 (3, 14)	0.73
**ISS**	22 (13, 34)	19 (9, 22)	34 (17, 43)*	0.02
**pH**	7.27 (708, 7.35)	7.35 (7.23, 7.42)	7.19 (6.91, 7.31)*	0.03
**Base excess (mEq/L)**	−2 (−3, 0)	−1 (−3, 0)	−3 (−10, 0)	0.23
**24 hr-total blood (U)**	5 (0, 28)	2 (0, 6)	28 (3, 41)*	0.006
**24 hr-total crystalloid (mL)**	2200 (1287, 4800)	1710 (1049, 7100)	2875 (1287, 4800)	0.82
**Length of stay (days)**	7 (2, 20)	5 (2, 17)	9 (2, 24)	0.87
**Mortality, n (%)**	8 (36%)	3 (27%)	5 (45%)	0.37

Median and inter-quartile range (IQR) values with the 25^th^ and 75^th^ percentiles in parentheses are reported. Asterisks (*) indicate statistical difference between Low and Normal COP groups (p < 0.05).

### Glycocalyx shedding, catecholamines and endothelial permeability

We observed significant increases in shedding of syndecan-1 and HA in trauma patients with low plasma COP compared to those with normal COP (Table 2). Median syndecan-1 levels were 6 times higher in patients with low COP compared to those with normal COP (222 ng/ml vs. 35 ng/ml, Table 2). Shed CS and HS chains were significantly higher in trauma patients compared to the healthy controls, but no detectable difference between the low and normal COP groups was observed. With regards to catecholamines, we observed almost a 10-fold increase in adrenaline levels in the low COP group compared to the normal COP group (p = 0.014), but no statistically significant increase in noradrenaline levels (p = 0.25) (Table 2). These increases in glycocalyx shedding and catecholamines corresponded to reduced TEER (i.e. increased permeability) as measured by ECIS, which reflect endothelial barrier integrity *in vitro*. HUVECs treated with 10% of plasma from the two groups reflected lower median endothelial resistances in the low COP group vs. the normal COP group (1067 Ωcm^2^ vs. 1101 Ωcm^2^, Table 2).

**Table 2:** **Summary of measured glycocalyx components, catecholamines and ECIS resistance**

	**Healthy controls (N = 5)**	**Normal COP (N = 11)**	**Low COP (N = 11)**	**p-value**
**Plasma COP (mmHg)**	21.2 (19.1, 21.3)	21.6 (16.2, 21.9)	12.4 (10.7, 13.9)^a,b^	<0.001
**Chondroitin sulfate (U/L)**	22.9 (22.5, 23.3)	32.3 (31.3, 33.7)^a^	32.7 (27.6, 33.9)^a^	0.003
**Heparan sulfate (ng/ml)**	133.9 (130.5, 138.3)	180.0 (176.4, 185.9)^a^	176.7 (151.5, 185.8)^a^	0.003
**Hyaluronic acid (ng/ml)**	627.6 (484.1, 753.1)	93.5 (49.0, 885.4)	380.9 (216.6, 682.4)^a,b^	0.035
**Syndecan-1 (ng/ml)**	31.6 ± 15.3*	34.6 (19.3, 43.1)	221.7 (88.3, 477.4)^a,b^	<0.001
**Adrenaline (ng/ml)**	72.1 ± 99.2^†^	90.5 (58.7, 226.9)	805.8 (659.3, 4319.3)^a,b^	0.014
**Noradrenaline (ng/ml)**	282 ± 454.7^†^	739.8 (189.5, 1006.2)	987.1 (352.3, 1187.4)	0.245
**TEER (Ωcm** ^**2**^ **)**	1066.3 (1057.8, 1074.8)	1101.6 (1089.6, 1115.5)	1067.3^b^ (1043.7, 1096.4)	0.016
**Normalized TEER**	1.30 (1.29, 1.31)	1.35 (1.33, 1.38)	1.31 (1.27, 1.33)	0.016

Median and IQR values are reported for each group. Kruskal-Wallis test was used to assess differences between groups (p-values shown). Multiple comparisons were also made: first comparing trauma patients to healthy controls and then comparing Low COP to Normal COP; ^a^indicates significant difference from healthy controls, ^b^indicates significant difference between Low and Normal COP groups (p < 0.05).

*Reference value for syndecan-1 levels in healthy volunteers was provided by Abcam.

^†^Adrenaline and noradrenaline healthy control values were obtained from Ostrowski et al. 2013 [16].

### Thrombin generation

**Figure 1:**
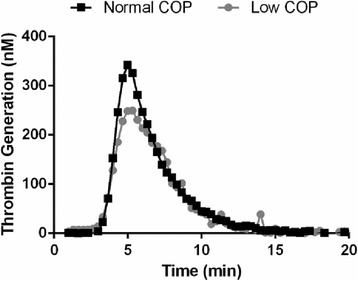
Representative thrombograms for trauma patients with Low (gray circle) vs. Normal (black square) plasma colloid osmotic pressure. Patients with low plasma COP have a marked reduction in peak thrombin generation (p < 0.05) compared to those with normal COP.

Patients with low plasma COP had shorter lag times, less ETP, reduced peak and similar ttPeak values compared to patients with normal COP. However, only peak thrombin generation was statistically significant (Table 3). A representative thrombogram from the CAT assay is illustrated in Figure 1. No remarkable differences in INR, aPTT or PT values were observed between groups. Platelet count was not statistically different between groups.

**Table 3:** **Summary of CAT parameters and conventional clinical coagulation tests**

	**Normal COP (N = 11)**	**Low COP (N = 11)**	**p-value**
**Lag time (min)**	3.83 (3.46, 4.83)	3.78 (3.33, 4.83)	0.77
ETP (nM*min)	1227 (1011, 1314)	1053 (1009, 1223)	0.53
**Peak (nM)**	325.4 (277.7, 352.2)	249.4 (177.4, 285.8)*	0.02
**ttpeak (min)**	5.7 (5.3, 6.7)	5.7 (5.3, 6.7)	0.97
**INR**	1.12 (1.07, 1.22)	1.19 (1.09, 1.59)	0.28
**aPTT (s)**	28.3 (26.5, 33.4)	32.8 (24.6, 43.1)	0.67
**PT (s)**	14.4 (13.9, 15.4)	15.1 (14.1, 18.9)	0.28

Median and IQR values are reported for each group. Peak thrombin generation is significantly lower in the Low COP group, as denoted by the asterisk (*).

### Correlations to patient outcomes and clinical parameters

Shed CS and HS were highly correlated (r = 0.95, p < 0.001) to each other, and HS was strongly associated with ISS (r = 0.60, p = 0.053). However, CS was not strongly associated with ISS (r = −0.21, p > 0.05). Hyaluronic acid exhibited the strongest correlation to ISS (r = 0.65, p = 0.03). ISS is a measure of anatomical injury, and so this correlation suggests that HA shedding is correlated to anatomical injury or more specifically increased tissue damage. Further, plasma COP was significantly associated with peak thrombin generation (r = 0.61, p = 0.03). More specifically, circulating syndecan-1 levels were inversely associated with ETP (r = −0.44, p = 0.048) and peak thrombin generation (r = −0.49, p = 0.02).

A multivariable linear regression for cellular permeability, as measured by ECIS, was developed. We observed that plasma from patients with low COP exhibited decreased resistance (i.e. more permeable), of 63 Ωcm^2^ on average (95% CI [47.8, 79.5 Ωcm^2^]), compared to plasma from patients with normal COP, while adjusting for age, gender, admission laboratory values, ISS and pre-hospital fluids. Furthermore, we determined that for every unit decrease in plasma COP, there is a 6.5 Ωcm^2^ drop in resistance, on average (95% CI [3.1, 10.05]). Plasma COP demonstrated the strongest association with TEER measurements, more than any of the individual EGL components (r = 0.64, p = 0.006).

A negative binomial regression for the total number of blood products transfused within the first 24 hours was developed. We observed that plasma COP, shed syndecan-1 and HA were significantly associated with increased number of blood transfusions within the first 24 hours. The incidence risk ratio for the low plasma COP group was 4.97 with 95% CI [1.12, 22.07], p = 0.035, while adjusting for age, gender, base excess, ISS and pre-hospital fluids. This means that patients who had low plasma COP were nearly 5 times more likely to receive blood transfusions than those with normal COP. As expected, patients who received pre-hospital fluids were more injured and thus required more blood transfusions (IRR: 3.1 with 95% CI [1.7, 5.6], p < 0.001).

## Discussion

Trauma patients with low plasma COP (< 16 mmHg) demonstrated increased shedding of EGL components, increased induced endothelial cell permeability *in vitro*, increased sympathoadrenal activation and hence circulating catecholamines, and reduced thrombin generation potential. The results from this small prospective study suggest that changes in the fluid mechanics, EGL and coagulopathy are associated with one another in severely injured trauma patients.

Similar to previous reports, we observed increased syndecan-1 shedding in severely injured trauma patients [5,17]. Syndecan-1 levels were associated not only with low plasma COP but also injury severity (i.e. ISS). Interestingly, HA was significantly higher in patients with low plasma COP, but there were no detectable differences in CS and HS levels between the low and normal COP groups. Together, the loss of syndecan-1 and HA along with the subsequent catecholamine response coincided with increased induced endothelial cell permeability *in vitro*. This data suggests that HA and its receptor (i.e. the CD44 receptor) may play a role in the maintenance of the EGL and vascular integrity during hemorrhagic shock. Rubio-Gayosso et al. have shown that effects of ischemia-reperfusion injury were partially prevented or reversed with intravascular infusion of exogenous HA [29]. Zeng et al. have reported the role of CS and HA in albumin binding and vascular permeability but the primary mechanisms of its release and capture remain unknown [30]. This study warrants further investigation of the role of HA and the CD44 receptor in maintaining vascular integrity during injury/hemorrhage and resuscitation.

Patients with low plasma COP had median peak thrombin concentrations less than 250 nM, while showing no other significant coagulopathy. Nevertheless, this cut-off for thrombin generation <250nM has been associated with worse outcomes and increased need for blood transfusions in a larger cohort of severely injured trauma patients [25]. A possible explanation for the reduced thrombin generation in the low COP group is that they are more injured and thus the thrombin is being consumed to achieve primary hemostasis. Patients with a combination of thrombin deficiency and increased EGL shedding could potentially be at the highest risk for morbidities such as acute lung injury, sepsis or multi-organ failure. These data suggest that developing resuscitation techniques that restore plasma proteins for improved plasma oncotic pressures, thrombin generation and ultimately the EGL could possibly improve outcomes for these severely injured patients.

The main limitation of this study is the relatively small sample size. We were able to conduct the ECIS measurements and additional glycocalyx component analysis on a small subset of our previously published cohort due to limited plasma sample volumes. Though smaller than the original cohort, the overall patient demographics of this subset group were consistent with the complete group, previously described by Rahbar et al. [24]. We also ensured to achieve a minimum of 75% power at the 0.05 significance level with this secondary subset analysis. Another limitation is that we measured circulating levels of the glycocalyx components from human plasma samples. While we know that the major source of these proteins is the endothelium, some of these glycocalyx components are also present on epithelial cells and some HA can be found on macrophages. Therefore it is difficult to ascertain the origin of the glycocalyx components in these human samples. In general, however, these components are considered to be glycocalyx injury biomarkers that are recognized by the research community. Future prospective studies are needed to evaluate new resuscitation techniques that can preserve the EGL during hemorrhage and resuscitation.

## Conclusions

The findings from this study reveal some of the interactions between the mechanical stimuli (i.e. plasma COP, hydrostatic pressures), endothelial barrier function, and coagulopathy in trauma patients. Plasma COP tends to correlate well not only with glycocalyx degradation, but also low thrombin generation potential. These interactions need to be studied in greater detail to reveal the key mechanisms of hemorrhagic shock and resuscitation, which will ultimately help patient care by developing improved targeted resuscitation strategies.
